# Genetic variants in histone modification regions are associated with the prognosis of lung adenocarcinoma

**DOI:** 10.1038/s41598-021-00909-z

**Published:** 2021-11-02

**Authors:** Hyo-Gyoung Kang, Yong Hoon Lee, Shin Yup Lee, Jin Eun Choi, Sook Kyung Do, Mi Jeong Hong, Jang Hyuck Lee, Ji Yun Jeong, Young Woo Do, Eung Bae Lee, Kyung Min Shin, Won Kee Lee, Sun Ha Choi, Hye won Seo, Seung Soo Yoo, Jaehee Lee, Seung Ick Cha, Chang Ho Kim, Sukki Cho, Sanghoon Jheon, Jae Yong Park

**Affiliations:** 1 Department of Biochemistry, School of Medicine, Kyungpook National University, Daegu, Republic of Korea; 2 Cell and Matrix Research Institute, School of Medicine, Kyungpook National University, Daegu, Republic of Korea; 3 Department of Internal Medicine, School of Medicine, Kyungpook National University, Daegu, Republic of Korea; 4 Lung Cancer Center, Kyungpook National University Chilgok Hospital, Daegu, Republic of Korea; 5 BK21 Plus KNU Biomedical Convergence Program, Department of Biomedical Science, Kyungpook National University, Daegu, Korea; 6 Department of Pathology, School of Medicine, Kyungpook National University, Daegu, Republic of Korea; 7 Department of Thoracic Surgery, School of Medicine, Kyungpook National University, Daegu, Republic of Korea; 8 Department of Radiology, School of Medicine, Kyungpook National University, Daegu, Republic of Korea; 9 Medical Research Collaboration Center in Kyungpook National University Hospital, Daegu, Republic of Korea; 10 Department of Medical Informatics, School of Medicine, Kyungpook National University, Daegu, Republic of Korea; 11 Department of Thoracic and Cardiovascular Surgery, Seoul National University School of Medicine, Seoul, Republic of Korea

**Keywords:** Cancer, Biomarkers

## Abstract

We investigated the association between genetic variants in the histone modification regions and the prognosis of lung adenocarcinoma after curative surgery. Potentially functional SNPs were selected using integrated analysis of ChIP-seq and RNA-seq. The SNPs were analyzed in a discovery set (n = 166) and a validation set (n = 238). The associations of the SNPs with overall survival (OS) and disease-free survival (DFS) were analyzed. A total of 279 SNPs were selected for genotyping. Among these, *CAPN1* rs17583C>T was significantly associated with better OS and DFS (*P* = 0.001 and *P* = 0.007, respectively), and *LINC00959* rs4751162A>G was significantly associated with worse DFS (*P* = 0.008). Luciferase assays showed a significantly lower promoter activity of *CAPN1* in the rs17583 T allele than C allele (*P* = 0.008), and consistently the CT + TT genotypes had significantly lower *CAPN1* expression than CC genotype (*P* = 0.01) in clinical samples. The rs4751162 G allele had higher promoter activity of *GLRX3* than A allele (*P* = 0.05). The motif analyses and ChIP-qPCR confirmed that the variants are located in the active promoter/enhancer regions where transcription factor binding occurs. This study showed that genetic variants in the histone modification regions could predict the prognosis of lung adenocarcinoma after surgery.

## Introduction

Carcinogenic process can be driven by the accumulation of epigenetic alterations as well as genetic alterations^1^. Epigenetics comprise heritable modifications to the chromatin that affect gene expression without altering DNA coding sequence, including DNA methylation, microRNA regulation, and histone modifications^2^. These epigenetic changes and their roles in human cancer have been actively studied for the past decades^2^. The knowledge of various epigenetic events has led to deeper understanding of the pathogenesis of cancer, and provided a clue to the discovery of prognostic biomarkers and novel therapeutic targets^1^. Histones are the central component of the nucleosomes, the fundamental building blocks of chromatin. The histone tails are subject to extensive posttranslational modifications such as methylation and acetylation among others, which can contribute to chromatin compaction, nucleosome dynamics, and transcriptional processes^3^. Dysregulation of these processes may lead to aberrant gene expression, which is frequently observed in human cancers^4^. Many studies have reported the role of genetic variations in the regulation of epigenome^5–8^, and their association with the risk and clinical outcomes of human cancers^9–12^.

Lung cancer is one of the most commonly diagnosed malignancies and is the leading cause of cancer-related death worldwide, with an average 5-year survival rate of 19%^13^. Lung adenocarcinoma comprises approximately 50% of all lung cancer^14^. Advanced understanding in molecular pathogenesis have enabled the identification of driver mutations and the development of personalized treatment options with clinically meaningful outcomes in metastatic lung adenocarcinoma^15,16^. In early-stage lung adenocarcinoma, many patients experience recurrence and even death after complete resection although surgery is the best treatment modality for cure. Even patients with the same stage are regarded to be at a variable risk of recurrence. However, reliable prognostic biomarkers are still not available. Therefore, deeper understanding in the molecular pathogenesis is essential to promote the development of prognostic biomarkers for building effective post-surgical strategies such as adjuvant therapy and follow-up to improve prognosis in early-stage NSCLC.

In this study, we hypothesized that potentially functional single nucleotide polymorphisms (SNPs) in histone modification regions may modulate the pathogenesis of lung cancer by regulating the expression of target genes, and consequently clinical outcomes of lung cancer. To test this hypothesis, we aimed to investigate the association between functional variants within or adjacent to genes with high expression in histone modification regions and the prognosis after surgery in lung adenocarcinoma.

## Results

### Patient characteristics and clinical outcomes

The baseline clinical and pathologic characteristics of patients in the discovery and validation cohorts and their association with OS and DFS are shown in Table 1. Median duration of follow-up was 30.4 (range, 3.6–67.1) months for the discovery cohort, and 27.4 (range, 1.0–66.7) months for the validation cohort. Pathologic stage was significantly associated with OS and DFS in both cohorts (log-rank *P* [*P*_L–R_] for OS = 0.01 and 3 × 10^–6^, and *P*_L–R_ for DFS = 4 × 10^–5^ and 3 × 10^–10^ in the discovery and the validation cohorts, respectively). In the discovery cohort, age was significantly associated with OS (*P*_L–R_ = 0.02), and adjuvant therapy was associated with DFS (*P*_L–R_ = 0.04). In the validation cohort, sex and smoking status were significantly associated with OS and DFS (*P*_L–R_ for OS = 0.04 and 0.01; and *P*_L–R_ for DFS = 0.03 and 0.01, respectively).

**Table 1:** Univariate analysis for overall survival and disease-free survival by clinicopathologic features in the discovery and validation cohorts.

Variables	Discovery cohort							Validation cohort						
	No. of cases	Overall survival			Disease-free survival			No. of cases	Overall survival			Disease-free survival		
		No. of death (%)^a^	5Y-OSR (%)^b^	Log-rank *P*	No. of event (%)^a^	5Y-DFSR (%)^b^	Log-rank *P*		No. of death (%)^a^	5Y-OSR (%)^b^	Log-rank *P*	No. of event (%)^a^	5Y-DFSR (%)^b^	Log-rank *P*
Overall	166	52 (31.3)	52		85 (51.2)	38		238	38 (16.0)	75		91 (38.2)	47	
**Age (years)**														
< 64	91	24 (26.4)	59	0.02	46 (50.6)	41	0.42	111	15 (13.5)	77	0.32	41 (36.9)	49	0.65
≥ 64	75	28 (37.3)	42		39 (52.0)	31		127	23 (18.1)	73		50 (39.4)	45	
**Sex**														
Male	92	34 (37.0)	47	0.11	52 (56.5)	33	0.28	129	25 (19.4)	70	0.04	56 (43.4)	37	0.03
Female	74	18 (24.3)	59		33 (44.6)	45		109	13 (11.9)	80		35 (32.1)	58	
**Smoking status**														
Never	75	18 (24.0)	63	0.11	35 (46.7)	42	0.53	132	16 (12.1)	81	0.01	45 (34.1)	56	0.01
Ever	91	34 (37.4)	44		50 (55.0)	35		106	22 (20.8)	65		46 (43.4)	35	
**Pack-years**^**c**^														
< 32	51	18 (35.3)	49	0.16	27 (52.9)	38	0.16	65	10 (15.4)	63	0.20	25 (38.5)	36	0.60
≥ 32	40	16 (40.0)	35		23 (57.5)	31		41	12 (29.3)	61		21 (51.2)	32	
**Pathologic stage**														
I	90	20 (22.2)	60	0.01	34 (37.8)	48	4 × 10^–5^	117	6 (5.1)	93	3 × 10^–6^	24 (20.5)	69	3 × 10^–10^
II–IIIA	76	32 (42.1)	43		51 (67.1)	25		121	32 (26.5)	55		67 (55.4)	24	
**Adjuvant therapy**^**d**^														
No	43	17 (39.5)	42	0.60	24 (55.8)	37	0.04	28	9 (32.1)	59	0.70	18 (64.3)	24	0.42
Yes	33	15 (45.5)	42		27 (81.8)	15		93	23 (24.7)	52		49 (52.7)	23	

^a^Row percentage.

^b^Five year-overall survival rate (5Y-OSR) and 5 year-disease free survival rate (5Y-DFSR), proportion of survival derived from Kaplan–Meier analysis.

^c^In ever-smokers.

^d^In pathologic stage II + IIIA.

### Identification of potentially functional SNPs using ChIP-seq and RNA-seq

The regulatory variants in histone modification regions may affect the expression of genes. To identify potentially functional variants in histone modification regions, we performed ChIP-seq with antibodies against two posttranslational modifications of histone H3 (H3K4me3 and H3K27ac) and RNA-seq using H2087 cell lines. Based on the ChIP-seq data for two histone marks, 31,582 SNPs located within H3K4me3 peaks and 34,591 within H3K27ac peaks were retrieved. And then, based on the NCBI SNP database (https://snpinfo.niehs.nih.gov/), 1654 SNPs within H3K4me3 peaks and 1229 within H3K27ac peaks, which were potentially functional and had minor allele frequency ≥ 0.1, were collected. Using RNA-seq we chose genes with high expression level (FPKM ≥ 100), and then 383 SNPs within or closest to the genes were extracted, of which 320 were identified as tag SNPs. Finally, the 279 SNPs were selected because 41 located within the overlap between the H3K4me3 peaks and H3K27ac peaks were duplicated.

### Associations between the SNPs and survival outcomes

**Figure 1:**
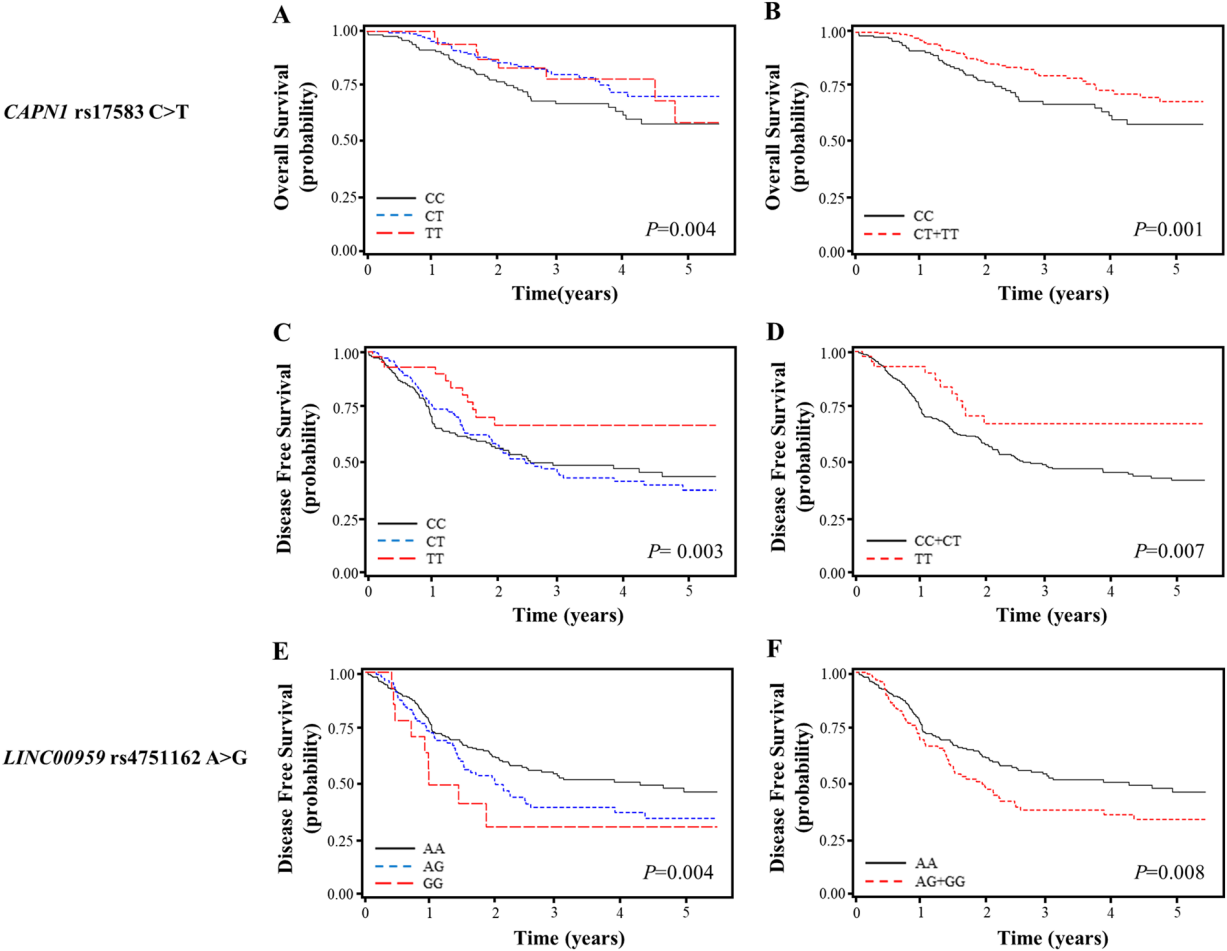
Overall survival (**A**, **B**) and disease-free survival (**C**, **D**) according to *CAPN1* rs17583C>T genotypes, and disease-free survival (**E**, **F**) according to *LINC00959* rs4751162A>G genotypes. The data were analyzed using SAS. *P* values in the multivariate Cox proportional hazard model.

Among the 279 SNPs genotyped, 184 were analyzed for the association study after excluding 10 with genotyping failure, 85 with deviation from the HWE (*P* < 0.05) or call rate < 90%. In the discovery set, 41 SNPs were significantly associated with clinical outcomes (Supplementary Table S1). Among 41 SNPs, two **(***CAPN1* rs17583C>T and *LINC00959* rs4751162A>G) were found to be significantly associated with survival outcomes in a validation set (Supplementary Table S2). In a combined analysis that adjusted for age, gender, smoking status, pathologic stage, and adjuvant therapy, *CAPN1* rs17583C>T was significantly associated with better OS and DFS (adjusted HR [aHR] = 0.49, 95% CI 0.32–0.75, *P* = 0.001, under a dominant model; aHR = 0.43, 95% CI 0.23–0.79, *P* = 0.007, under a recessive model, respectively), and *LINC00959* rs4751162A>G exhibited worse DFS (aHR = 1.53, 95% CI 1.11–2.09, *P* = 0.008, under a dominant model) (Fig. 1 and Table 2).

**Table 2:** Overall and disease-free survival according to genotypes of two polymorphisms in the combined cohorts.

Polymorphism/genotypes^e^	No. of cases (%)^a^	Overall survival				Disease-free survival			
		No. of events (%) ^b^	5Y-OSR (%)^c^	HR (95% CI)^d^	*P*^d^	No. of events (%)^c^	5Y-DFSR (%)^c^	HR (95% CI)^d^	*P*^d^
**rs17583**									
CC	173 (43.8)	48 (27.8)	58	1.00		79 (45.7)	43	1.00	
CT	178 (45.1)	31 (17.4)	71	0.48 (0.31–0.76)	0.002	80 (44.9)	37	0.80 (0.58–1.10)	0.16
TT	44 (11.1)	8 (18.2)	59	0.51 (0.24–1.09)	0.08	11 (25.0)	67	0.38 (0.20–0.71)	0.003
Dominant	222 (56.2)	39 (17.6)	69	0.49 (0.32–0.75)	0.001	91 (41.0)	42	0.71 (0.52–0.96)	0.03
Recessive	351 (88.9)	79 (22.5)	65	0.74 (0.35–1.54)	0.42	159 (45.3)	41	0.43 (0.23–0.79)	0.007
Codominant				0.60 (0.42–0.85)	0.004			0.69 (0.54–0.88)	0.003
**rs4751162**									
AA	273 (68.9)	58 (21.3)	66	1.00		107 (39.2)	47	1.00	
AG	108 (27.3)	28 (25.9)	60	1.30 (0.82–2.05)	0.27	55 (50.9)	35	1.45 (1.04–2.01)	0.03
GG	15 (3.8)	2 (13.3)	60	0.63 (0.15–2.63)	0.53	9 (60.0)	31	2.23 (1.11–4.48)	0.03
Dominant	123 (31.1)	30 (24.4)	60	1.21 (0.78–1.89)	0.40	64 (52.0)	35	1.53 (1.11–2.09)	0.008
Recessive	381 (96.2)	86 (22.6)	64	0.59 (0.14–2.45)	0.47	162 (42.5)	43	2.02 (1.01–4.04)	0.05
Codominant				1.09 (0.75–1.58)	0.65			1.47 (1.13–1.90)	0.004

^a^Column percentage.

^b^Row percentage.

^c^Five year-overall survival rate (5Y-OSR) and 5 year-disease free survival rate (5Y-DFSR), proportion of survival derived from Kaplan–Meier analysis.

^d^Hazard ratios (HRs), 95% confidence intervals (CIs) and corresponding *P*-values were calculated using multivariate Cox proportional hazard models, adjusted for age, gender, smoking status, pathologic stage and adjuvant therapy.

^e^Genotype failure: nine cases for the rs17583 and eight cases for the rs4751162.

### Effect of rs17583 C>T and rs4751162 A>G on promoter activity and mRNA expression

**Figure 2:**
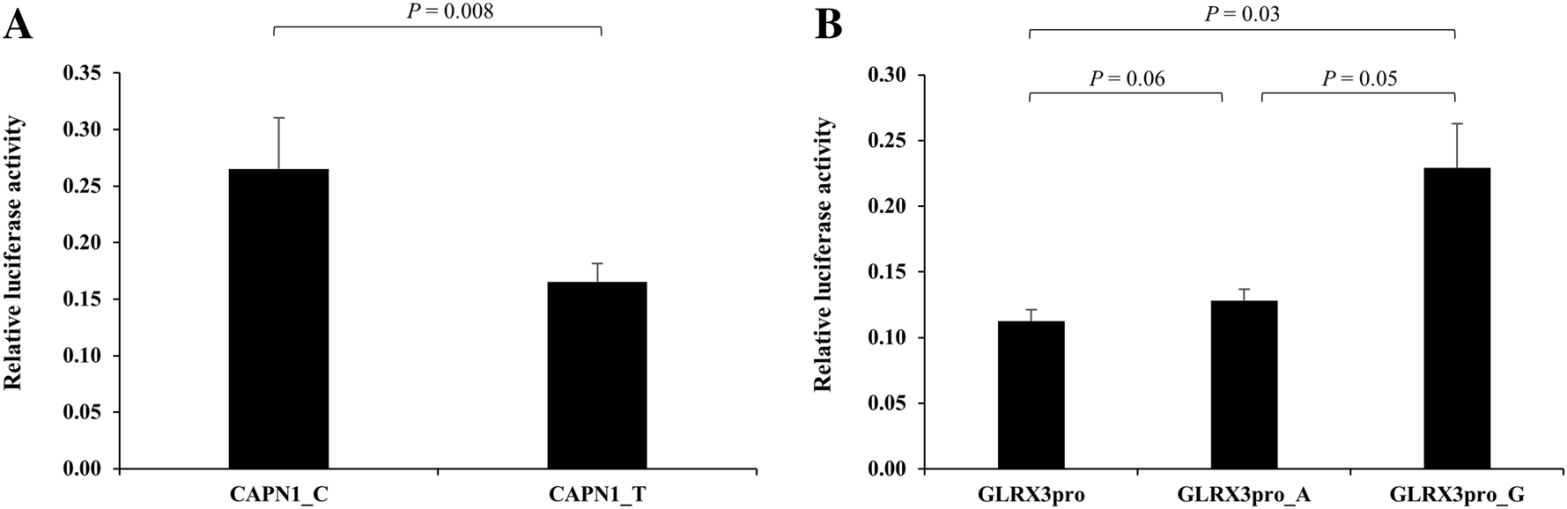
The transcription activity according to alleles of *CAPN1* rs17583C>T and *LINC00959* rs4751162A>G measured by luciferase reporter assay. The H1703 cells were transfected with pGL3-*CAPN1*_C and pGL3-*CAPN1*_T for rs17583 (**A**), and pGL3-*GLRX3*pro, pGL3-*GLRX3*pro_A, or pGL3-*GLRX3*pro_G for rs4751162 (**B**), respectively. The data were analyzed using Excel. Each bar represents mean ± SEM of luciferase activity normalized to Renilla luciferase activity. Experiments were performed in triplicate. *P* value, Student's t-test.

**Figure 3:**
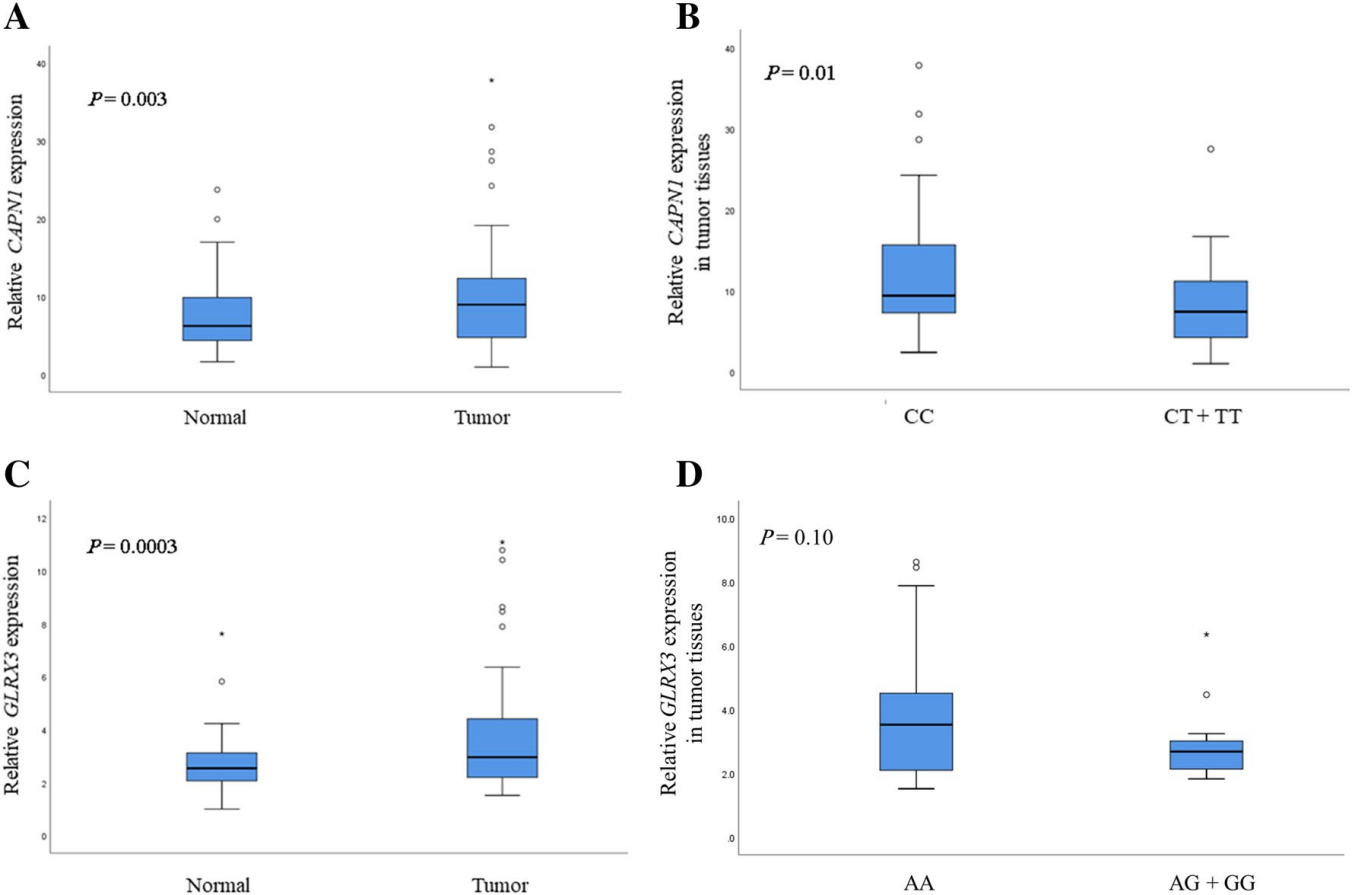
The mRNA expression levels of *CAPN1* and *GLRX3* in tumor and corresponding non-malignant lung tissues (**A**, n = 73 and **C**, n = 55), and *CAPN1* and *GLRX3* mRNA expression according to *CAPN1* rs17583C>T (32CC, 29CT, and 8TT) and *LINC00959* rs4751162A>G (32AA, 15AG, and 1GG) genotypes (**B**, **D**). The data were analyzed using SPSS. The *P*-value was calculated using Student’s t-test.

The rs17583C>T is located in the promoter region of *CAPN1*, and the rs4751162A>G is located in the intron of *LINC00959*. The rs4751162 resides in an enhancer which is 26 kb apart from *GLRX3* and expected to regulate its expression. To verify the functional relevance of the two genetic variants, we investigated whether rs17583C>T and rs4751162A>G regulate the promoter activity of the *CAPN1* and *GLRX3* gene, respectively. The promoter assays showed that the *CAPN1* rs17583 T allele had significantly lower promoter activity than the C allele (Fig. 2A, P = 0.008). The *LINC00959* rs4751162 G allele had significantly higher *GLRX3* promoter activity than the A allele (Fig. 2B, P = 0.05). Quantitative RT-PCR showed that the relative *CAPN1* expression was significantly higher in tumor tissue than in normal lung tissue (Fig. 3A, P = 0.003). In tumor tissues, *CAPN1* expression was significantly lower in CT + TT than in CC genotype (Fig. 3B, P = 0.01). The expression of *GLRX3* was also significantly higher in tumor tissue than in normal tissue (Fig. 3C, P = 0.0003). *GLRX3* expression was not significantly different among genotypes (Fig. 3D). There was no significant association between mRNA expression level and the survival outcomes (data not shown).

### ChIP-qPCR assays to confirm histone modification and transcription factor binding in two SNP-containing regions

**Figure 4:**
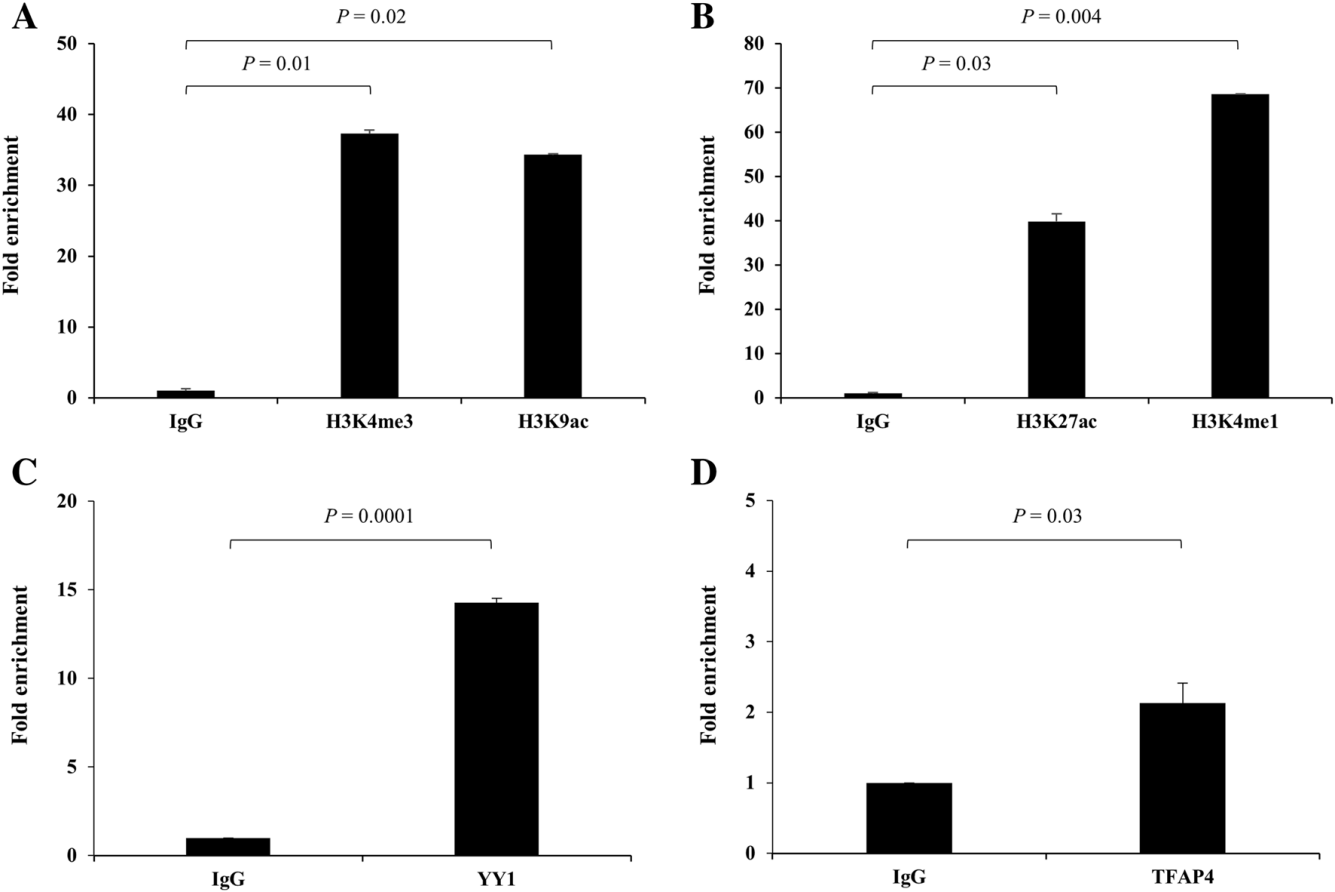
ChIP-qPCR analysis of H3K4me3 and H3K9ac (**A**) and YY1 (**C**) binding at *CAPN1* rs17583, and H3K27ac and H3K4me1 (**B**) and TFAP4 (**D**) binding at *LINC00959* rs4751162. The data were analyzed using Excel. Each bar represents mean ± SEM from three independent experiments carried out in triplicate. *P* values by Student’s t-test.

To confirm whether the two genetic variants are located in functional promoter or enhancer with active histones, we performed ChIP-qPCR assays using antibodies against H3K4me3, H3K27ac, H3K9ac, and H3Kme1. The rs17583-containing region was associated with strong enrichment of H3K4me3 and H3K9ac which mark active promoters (Fig. 4A), and the rs4751162-containing region showed strong enrichment of H3K27ac and H3Kme1 which mark active enhancers (Fig. 4B). Next, motif analyses were performed to predict transcription factors which bind to the SNP regions. As a result of aligning with known motifs using TOMTOM tool in the MEME Suite web server^17^, the rs17583 and rs4751162 are located in the binding motifs of YY1 and TFAP4, respectively (Supplementary Fig. S1). We performed ChIP assays with antibodies to YY1, TFAP4 or with IgG as a control. The rs17583 and rs4751162 regions showed significant enrichment of YY1 and TFAP4, respectively (Fig. 4C,D). The YY1 was predicted to bind to rs17583 C allele more efficiently than to T allele, and TFAP4 was predicted to bind to rs4751162 G allele more efficiently than to A allele, suggesting the alleles with increased gene expression had preferred transcription factor binding (Supplementary Fig. S1).

## Discussion

In this study, we investigated the association between genetic variation in histone modification regions and the prognosis in lung adenocarcinoma. We found that two genetic variants, *CAPN1* rs17583C>T and *LINC00959* rs4751162A>G, in the histone modification regions were associated with the survival outcomes of patients with lung adenocarcinoma who underwent curative surgery. Functional analyses showed significant difference in the promoter activity and expression level of *CAPN1* and *GLRX3* genes according to genotypes, supporting the association between the variants and the survival outcomes. The motif analyses and ChIP-qPCR confirmed that the variants are located in the active promoter/enhancer regions where transcription factor binding occurs, elucidating epigenetic regulatory mechanisms of gene expression by the variants. These results suggested a potential role of the two genetic variants in the pathogenesis of lung adenocarcinoma, and their usefulness as prognostic biomarkers.

Calpains (CAPNs) are a family of calcium-dependent cysteine proteinases involved in a variety of cellular processes including remodeling of cytoskeletal/membrane attachments, signal transduction, and apoptosis^18^. CAPN1 and CAPN2 are the two isoforms of the ubiquitous calpain, which are heterodimers of a large catalytic subunit encoded by *CAPN1* and *CAPN2*, respectively, and a regulatory subunit (CAPN4) encoded by *CAPNS1*^19^. CAPNs could cause limited cleavage or functional modulation of various substrates that act as metastatic mediators, and several studies have suggested that it plays a significant role in tumor migration and invasion^20^. Only a few studies have addressed the role of CAPNs in lung cancer. It has been reported that CAPN2 might promote lung cancer progression by activating EGFR/pAKT signaling pathway, and also contribute to the resistance to paclitaxel or EGFR-TKI^21,22^. Another study suggested that overexpression of CAPN4 was an independent prognostic factor in patients with NSCLC, and could enhance the invasive potential of lung cancer cells by upregulating the expression of matrix metalloproteinase 2^23^. In the present study, the *CAPN1* rs17583C>T was associated with significantly better clinical outcomes. The *CAPN1* expression was significantly higher in lung adenocarcinoma than normal lung. Functional analyses showed that rs17583C-to-T change was associated with decreased *CAPN1* mRNA expression in clinical samples and significantly decreased *CAPN1* promoter activity in i*n vitro* promoter assays. The ChIP-qPCR confirmed that *CAPN1* rs17583C>T is located in an active promoter with YY1 binding. These results suggested a tumor promoting function of *CAPN1* in lung adenocarcinoma, and provided evidence of the functional relationship between the genetic variant and the better clinical outcomes.

GLRX3 is an antioxidant enzyme, one of the intracellular redox-regulating molecules that contribute to maintaining cellular redox homeostasis, and is known to play an important role in cellular signal transduction in response to stress signals by reactive oxygen species^24^. Although the complicated roles of *GLRX3* in cancer remain poorly understood, overexpression of *GLRX3* was ascertained in several types of malignancy, such as nasopharyngeal carcinoma (NPC), oral squamous cell carcinoma (OSCC), colon cancer, and lung cancer^25–27^. Studies on NPC and OSCC have reported in common that knockdown of *GLRX3* inhibited cell proliferation and decreased the migration and invasion capacity of cancer cells by suppressing the epithelial-mesenchymal transition, indicating the essential roles of GLRX3 in cancer progression^25,26^. In agreement with these results, our unpublished data indicated that knockdown of *GLRX3* inhibited proliferation, migration, and invasion capacity of lung cancer cells. In this study, *LINC00959* rs4751162A>G was associated with worse DFS. *GLRX3* expression was significantly higher in tumor tissues than in normal lung, and rs4751162 G allele correlated with a significantly higher promoter activity of *GLRX3* than the A allele. Motif analyses predicted that the rs4751162 is located in the binding motif of TFAP4, and the ChIP-qPCR confirmed the SNP was located in an active enhancer with TFAP4 binding. These results suggested a potentially oncogenic role of *GLRX3*. However, further investigation is needed to understand the role of *CAPN1* and *GLRX3* in the pathogenesis of lung adenocarcinoma and the biological mechanisms of the association between the genetic variants and clinical outcomes.

In this study, only two out of 41 SNPs were replicated in the validation cohorts. Difference in clinical characteristics of the patients between the discovery and validation cohorts, which could be a possible cause of replication failure, is a limitation of the retrospective multicenter studies. However, all the clinical variables were adjusted for in the multivariate analyses. More importantly, because the discovery study with a relatively small sample size was an exploratory study for which type II errors should be considered, we did not perform multiple testing corrections for the associations, which may have resulted in false positive associations leading to the replication failure of the associations in the validation study. In addition, the modest sample size of the validation cohort may not have optimal statistical power for replicating the associations. In this study, the observed *P* values did not reach a more stringent level of statistical significance to avoid false positive associations arising from multiple comparisons. Future studies with larger number of patients are required to validate our results. Nevertheless, the design of two-stage independent cohorts for the discovery and validation sets is a major strength of this study, which could reduce false-positive findings from the genetic association study^28,29^. The two SNPs were significantly associated with clinical outcomes in both independent cohorts, and the association had similar effect size with the same direction. In addition, the association showed higher level of significance in the combined analysis including larger population, supporting the credibility of the association. Functional analyses further supported the plausibility of our findings.

In summary, the current study demonstrated that the two genetic variants, *CAPN1* rs17583C>T and *LINC00959* rs4751162A>G, was associated with survival outcomes of patients with lung adenocarcinoma after surgical resection. Our results suggest that *CAPN1* and *GLRX3* may play important roles in the pathogenesis of lung adenocarcinoma, and that the variants may be useful in predicting the prognosis of lung adenocarcinoma after surgery, thereby helping to refine therapeutic decisions for better clinical outcomes in NSCLC. Future studies are warranted to validate our results in a larger population with diverse ethnicity.

## Methods

### Study population

A total of 404 patients with available genomic DNA samples, who were diagnosed with pathologic stages I, II, or IIIA (micro-invasive N2) NSCLC after curative surgical resection, were enrolled in this study. Discovery cohort comprised 166 patients whose diagnosis was made at Kyungpook National University Hospital (KNUH) between September 2001 and August 2009, and validation cohort consisted of 238 patients diagnosed with NSCLC at Seoul National University Bundang Hospital (SNUBH) between June 2005 and May 2012. All patients in this study were of Korean ethnicity. Genomic DNA samples extracted from peripheral blood lymphocytes of the patients were provided by the National Biobank of Korea, KNUH, which is supported by the Ministry of Health, Welfare and Family Affairs. Written informed consent was obtained from all patients before surgery. This study was approved by the Institutional Review Boards of the Kyungpook National University Chilgok Hospital and Seoul National University Bundang Hospital (Approval No. KNUCH 2017-07-012). All experiments were performed in accordance with relevant guidelines and regulations.

### Cell culture and antibodies

H2087 cells were obtained from the American Type Culture Collection (ATCC) and H1703 cells were purchased from Korean Cell Line Bank (KCLB), Seoul, Korea. Antibodies used in this study include anti-Histone H3 antibodies (ab8580, ab4441, ab4729 and ab8895), and anti-GLRX3 antibody (ab226396) from Abcam (Cambridge, UK), anti-YY1 antibody (46395) from Cell Signaling Technology (Danvers, MA, USA), and anti-TFAP4 antibody (sc-166216X) from Santa Cruz Biotechnology (Dallas, TX, USA). Cells were cultured at 37 °C in a humidified atmosphere with 5% CO_2_ in Corning RPMI medium (Corning Inc., Corning, NY, USA) supplemented with 10% Corning Fetal Bovine Serum (Corning Inc., Corning, NY, USA), and 100 U/ml penicillin and 100 mg/ml streptomycin.

### Chromatin immunoprecipitation (ChIP)-sequencing

ChIP assays were performed using the Pierce Magnetic ChIP kit (Thermo Fisher Scientific, Waltham, MA, USA), according to the manufacturer’s protocol. H2087 cells were crosslinked with 1% formaldehyde for 10 min, and the crosslinking was inactivated by 0.125 M glycine for 5 min at room temperature. Cells were washed with cold 1xPBS twice. The cells were lysed, sonicated to shear DNA. To immunoprecipitate protein/chromatin complexes, the diluted supernatants were incubated with 10 μg of H3K4me3 or H3K27ac antibody overnight, and then incubated for 2 h after adding 50 μl of agarose/protein A or G beads. Ten percent of the diluted supernatants were saved as “input” for normalization. Several washing steps were followed by protein digestion using proteinase K. Reverse crosslinking was carried out at 65 °C. DNA was subsequently purified. ChIPSeq library preparation was performed with TruSeq ChIP Library Preparation Kit (Illumina, San Diego, CA, USA). Sequencing was performed on an Illumina HiSeq4000. Sequence reads for each sample were aligned to the human genome using Bowtie^30^. The reference genome sequence of Homo sapiens (hg19) and annotation data were downloaded from the UCSC table browser (http://genome.uscs.edu). Peaks were called in the aligned sequence data using a model-based analysis of ChIP-seq (MACS2 version 2.1.0) (https://bioweb.pasteur.fr/packages/pack@macs@2.1.0)^31^. ChIPseeker (version 1.6.6) (http://www.bioconductor.org/packages/release/bioc/html/ChIPseeker.html)^32^, a bioconductor package within the statistical programming environment R to facilitate batch annotation of enriched peaks identified from ChIP-seq data, was used to identify nearby genes and transcripts from the peaks obtained from MACS2.

### RNA-sequencing

Total RNAs from H2087 cells were isolated using TRIzol (Invitrogen, Carlsbad, CA, USA). Sequencing was performed on an Illumina HiSeq4000 and aligned the processed reads to the Homo sapiens (hg19) using HISAT v2.0.5^33^. Transcript assembly and abundance estimation was performed using StringTie v1.3.3b^34,35^. It provides the relative abundance estimates as FPKM values (Fragments Per Kilobase of exon per Million fragments mapped) of transcript and gene expressed in each sample. FPKM values have already been normalized with respect to library size.

### SNP selection and genotyping

We conducted an integrated analysis of ChIP-seq and RNA-seq for the SNP selection. As a result of the ChIP-seq using H2087 cells, SNPs within H3K4me3 and H3K27ac peak regions were selected. Next, using the FuncPred utility for functional SNP prediction in the SNPinfo web server (https://snpinfo.niehs.nih.gov/), potentially functional variants with minor allele frequency ≥ 0.1 based on the HapMap JPT data were collected after excluding those in linkage disequilibrium (*r*^2^ ≥ 0.8). And then, using RNA-seq we chose genes with high expression level (FPKM ≥ 100), and then SNPs within or closest to the genes were extracted. Genotyping was performed using iPLEX Assay and MassARRAY System (Agena Bioscience, San Diego, CA, USA). Approximately 5% of the samples were randomly selected to be genotyped again by a different investigator, by a restriction fragment length polymorphism assay, and the results were 100% concordant.

### Promoter-luciferase constructs and luciferase assay

We evaluated the effect of the rs17583C>T or rs4751162A>G on the activity of the promoter of *CAPN1* or *GLRX3* genes by luciferase reporter assay. The rs17583C>T of *CAPN1* gene is located in the region of H3K4me3 peak, which marks active promoters, in *CAPN1* gene promoter. The 378 bp fragment including rs17583C>T was synthesized by polymerase chain reaction from human genomic DNA and cloned into *XhoI*/*HindIII* site of the pGL3-basic vector (Promega, Madison, WI, USA). The correct sequences of all clones were verified by DNA sequencing. The rs4751162A>G is located in the region of H3K27ac peak, which is an activation mark of enhancers, in the intron region of *LINC00959* gene. The SNP is expected to regulate expression of *GLRX3* gene because it resides 26 kb downstream of *GLRX3* gene, although they both are on the chromosome 10. The promoter region of *GLRX3* (− 980 to + 38 bp, the transcriptional start site is designated as + 1) was synthesized by polymerase chain reaction from human genomic DNA and cloned into *XhoI/NcoI* site of the pGL3-promoter vector (Promega, Madison, WI, USA) to generate pGL3-*GLRX3*pro. Two fragments including rs4751162A or rs4751162G allele of rs4751162A>G were amplified from genomic DNA sample and the 283 bp products were cloned into *BamHI/SalI* site of the pGL3-*GLRX3*pro, respectively, to generate pGL3-*GLRX3*pro_A and pGL3-*GLRX3*pro_G. The cloning PCR primers were listed Supplementary Table S3. All constructs were verified by direct sequencing before use. The H1703 cells were transfected with 200 ng of each plasmid DNA (pGL3-*CAPN1*_C and pGL3-*CAPN1*_T for rs17583, and pGL3-*GLRX3*pro, pGL3-*GLRX3*pro_A, or pGL3-*GLRX3*pro_G for rs4751162) and 2 ng of pRL-SV40 Vector (Promega, Madison, WI, USA) using Effectene transfection reagent (Qiagen, Hilden, Germany) according to manufacturer’s protocol. The cells were collected 48 h after transfection. Luciferase activity was measured using the Dual-Luciferase Reporter Assay System (Promega, Madison, WI, USA). Firefly luciferase activity measurements were normalized with respect to pRL-SV40 *Renilla* luciferase activity to correct for variations in transfection efficiency. All experiments were performed in triplicate.

### RNA preparation and quantitative reverse transcription-PCR (qRT-PCR)

*CAPN1* and *GLRX3* mRNA expression was examined by qRT-PCR. Total RNAs from tumors and paired non-malignant lung tissues (n = 73) were isolated using TRIzol (Invitrogen, Carlsbad, CA, USA). Real-time PCR was performed using a LightCycler 480 (Roche Applied *GLRX3* expression Science, Mannheim, Germany) with QuantiFast SYBR Green PCR Master Mix (Qiagen, Hilden, Germany). The real-time PCR primers for *CAPN1*, *GLRX3* and *β-actin* genes were listed in Supplementary Table S3. Each sample was run in duplicate. Relative target gene mRNA expression was normalized to that of *β-actin* expression and then evaluated using the 2^−ΔΔ*C*t^ method^36^.

### ChIP-quantitative PCR (qPCR) assay

Chromatin from H2087 cells was immunoprecipitated with the Pierce Magnetic ChIP kit (Thermo Fisher Scientific, Waltham, MA, USA), using 10 μg anti-H3K4me3, anti-H3K9ac, anti-H3K27ac, anti-H3Kme1, anti-YY1, and anti-TFAP4 antibodies and 2 μg normal rabbit IgG antibodies per immunoprecipitation reaction. Immunoprecipitated chromatin was subjected to real-time qPCR using SYBR Green PCR Master Mix (Qiagen, Hilden, Germany). The ChIP-qPCR primers were listed Supplementary Table S3. The qPCR was performed as follows: 95 ℃ 10 min, 45 cycles of 95 ℃ 15 s, 60 ℃ 1 min. ChIP-qPCR enrichment analysis were performed by Comparative Ct method. Each samples were normalized to the input and the fold difference between sample and IgG was calculated using 2(− ΔΔCt)^37^.

### Enriched motif analysis

We identified the transcription factor binding motif enriched in the regions containing rs17583C>T or rs4751162 A>G. Motifs were analyzed using TOMTOM, a motif database scanning algorithm, of the MEME Suite web server^17^ for comparison against Human and Mouse^38^ and the SwissRegulon databases of known transcription factor motifs^39^.

### Statistical analyses

Hardy–Weinberg equilibrium was evaluated by a goodness-of-fit χ^2^ test with 1 degree of freedom. Overall survival (OS) was measured from the date of surgery to the date of death or the last follow-up. Disease-free survival (DFS) was calculated from the date of surgery until first evidence of disease recurrence or last date of follow up for patients who were free of disease. Estimated survival rate was calculated using the Kaplan–Meier method. Log-rank test was used to compare the difference in OS and DFS across different genotypes. Multivariate Cox proportional hazards models were used to estimate the hazard ratio (HR) and 95% confidence intervals (CI) after adjusting for age (< 64 years vs ≥ 64 years), gender (male vs female), smoking status (never vs ever), pathological stage (I vs II–IIIA), and adjuvant therapy (yes vs no). Statistical analyses were carried out using Statistical Analysis System for Windows, version 9.4 (SAS Institute, Cary, NC, USA), Statistical Package for the Social Sciences (SPSS) 25.0 (IBM Corp., Armonk, NY, USA), and Microsoft Excel (Microsoft Corp., Redmond, WA, USA).

## Supplementary Information

Supplementary Information.

## Data Availability

The datasets for the RNA-seq and ChIP-seq (H3K4me3, H3K27ac) have been deposited and are available at Gene Expression Omnibus (GEO accession no. GSE182385) (https://www.ncbi.nlm.nih.gov/geo/query/acc.cgi?acc=GSE182385).
