# Supplementary material for: Genetic variants in histone modification regions are associated with the prognosis of lung adenocarcinoma
Source: Sci Rep. 2021 Nov 2;11:21520. doi: 10.1038/s41598-021-00909-z (PMC8563968; doi:10.1038/s41598-021-00909-z)
Supplement: Supplementary file 1 — Supplementary Information. [file 41598_2021_909_MOESM1_ESM.pdf]

Genetic variants in histone modification regions are associated with the prognosis of lung adenocarcinoma

Hyo-Gyoung Kang[1,2,*], Yong Hoon Lee [3,*], Shin Yup Lee[3,4], Jin Eun Choi[1,2], Sook Kyung Do[1,2], Mi Jeong Hong[1,2], Jang Hyuck Lee[1,5], Ji Yun Jeong[6], Young Woo Do[4,7], Eung Bae Lee[4,7], Kyung Min Shin[8], Won Kee Lee[9,10], Sun Ha Choi[3,4], Hye won Seo[3], Seung Soo Yoo[3,4], Jaehee Lee[3], Seung Ick Cha[3], Chang Ho Kim[3], Sukki Cho[11], Sanghoon Jheon[11], Jae Yong Park[1,2,3,4,5]

*These authors contributed equally to this work.

Corresponding authors

Jae Yong Park, MD, PhD, Lung Cancer Center, Kyungpook National University Chilgok Hospital, 807, Hoguk-ro, Buk-gu, Daegu 41404, Korea; Tel: +82-53-200-2631; Fax: +82-53-200-2027, E-mail: jaeyong@knu.ac.kr

Shin Yup Lee, MD, PhD, Lung Cancer Center, Kyungpook National University Chilgok Hospital, 807, Hoguk-ro, Buk-gu, Daegu 41404, Korea; Tel: +82-53-200-2632; Fax: +82-53-200-2027, E-mail: shinyup@knu.ac.kr

Supplementary Information

Supplementary Figure S1, Supplementary Table S1, S2 and S3

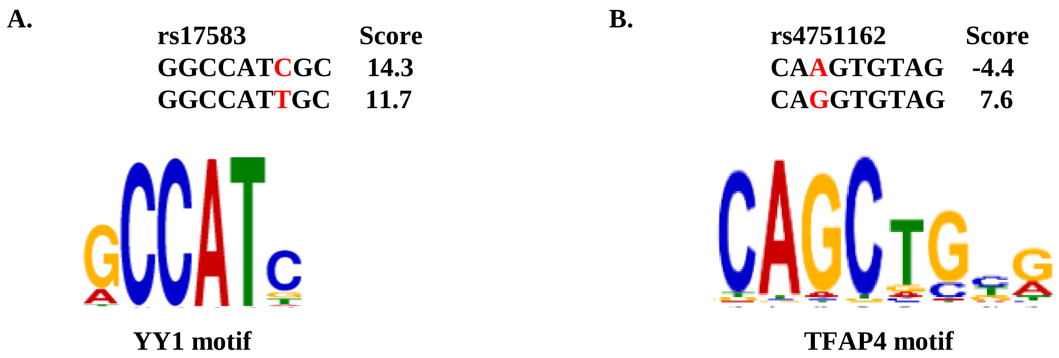

Supplementary Figure S1. Matched motifs of YY1 and TFAP4, and the predicted binding scores of rs17583 and rs4751162. The rs17583 and rs4751162 are located in the binding motifs of YY1 and TFAP4, respectively. Predicted binding scores using the HaploReg database*

*Lucas D Ward, Manolis Kellis. HaploReg: a resource for exploring chromatin states, conservation, and regulatory motif alterations within sets of genetically linked variants. Nucleic Acids Res. 2012;40:D930-4.

Supplementary Table S1. List of genotyped SNPs and analysis for overall survival and disease-free survival in the discovery study.

Polymorphism/Gene[a]			Genotype		Overall survival, *P-value* [b]			Disease Free survival, *P-value* [b]		
ID No.	Alleles	Location	MAF	HWE-*p*	Dominant	Recessive	Codominant	Dominant	Recessive	Codominant
rs17583	C>T	O	0.34	0.53	0.001	0.96	0.02	0.15	0.24	0.09
rs4751162	A>G	C	0.16	0.47	0.23	0.56	0.21	0.04	0.92	0.06
rs1529959	G>A	O	0.49	0.58	0.06	0.71	0.34	0.03	0.61	0.33
rs2601006	C>T	O	0.26	0.24	0.75	0.10	0.30	0.65	0.02	0.15
rs757228	G>A	O	0.36	0.45	0.11	0.94	0.21	0.03	0.67	0.07
rs11188272	G>C	C	0.39	0.18	0.23	0.03	0.83	0.71	0.002	0.19
rs4669	T>C	O	0.39	0.05	0.03	0.69	0.19	0.07	0.39	0.45
rs6049283	A>C	O	0.21	0.07	0.04	0.19	0.19	0.46	0.11	0.99
rs1205366	C>T	O	0.15	0.38	0.03	0.88	0.05	0.16	0.28	0.11
rs17528989	G>A	C	0.14	0.07	0.89	0.20	0.60	0.47	0.01	0.15
rs2425047	A>C	O	0.19	0.28	0.78	0.02	0.56	0.87	0.16	0.75
rs1136267	C>A	O	0.21	0.11	0.07	1.00	0.11	0.02	1.00	0.04
rs1803956	T>C	O	0.41	0.14	0.62	0.02	0.11	0.64	0.03	0.16
rs34087264	C>T	O	0.39	0.76	0.47	0.11	0.69	0.93	0.02	0.22
rs3896021	G>T	C	0.52	0.13	0.10	0.89	0.30	0.01	0.17	0.01
rs13817	G>A	C	0.35	0.48	0.05	0.11	0.02	0.89	0.68	0.77
rs2234216	T>C	O	0.36	0.30	0.92	0.003	0.13	0.53	0.23	0.90
rs6547618	G>A	C	0.37	0.67	0.33	0.03	0.06	0.99	0.15	0.43
rs6601178	G>A	C	0.47	0.61	0.01	0.05	0.01	0.02	0.25	0.03
rs2270625	C>G	O	0.26	0.21	0.01	0.20	0.01	0.31	0.79	0.37
rs17122154	A>G	O	0.44	0.39	0.47	0.59	0.88	0.04	0.43	0.07
rs2548551	T>G	C	0.13	0.41	0.22	0.25	0.16	0.67	0.0003	0.22
rs10139029	A>G	O	0.19	0.09	0.03	0.53	0.05	0.78	0.82	0.75
rs665306	T>C	O	0.34	0.75	0.08	0.08	0.03	0.71	0.84	0.85
rs1010222	G>A	O	0.13	0.71	0.34	0.11	0.20	0.84	0.01	0.44
rs2271446	C>T	O	0.27	0.46	0.04	0.26	0.05	0.07	0.50	0.10
rs3825625	C>T	O	0.15	0.85	0.03	0.63	0.04	0.11	0.51	0.10
rs7513326	G>A	C	0.43	0.10	0.12	0.01	0.01	0.55	0.04	0.14
rs772254	C>T	O	0.21	0.14	0.02	0.39	0.03	0.09	0.29	0.08
rs11668444	G>T	C	0.18	0.30	0.09	0.02	0.02	0.38	0.29	0.27
rs1279738	C>G	C	0.46	0.63	0.001	0.004	0.0002	0.02	0.04	0.01
rs2075588	A>C	O	0.29	0.19	0.09	0.27	0.06	0.31	0.03	0.11
rs3806192	G>C	O	0.19	0.05	0.46	0.99	0.57	0.01	0.98	0.03
rs843346	C>T	O	0.37	0.13	0.94	0.01	0.09	0.65	0.01	0.11
rs1242095	A>C	O	0.36	1.00	0.55	0.03	0.15	0.07	0.01	0.01
rs2292807	T>C	O	0.44	0.94	0.70	0.03	0.29	0.31	0.16	0.88
rs6679	T>C	C	0.43	0.56	0.40	0.64	0.75	0.04	0.38	0.06
rs11809443	T>C	C	0.22	0.28	0.50	0.05	0.15	0.87	0.25	0.56

rs2271541	T>G	O	0.41	0.68	0.03	0.11	0.51	0.06	0.17	0.57
rs3813521	C>T	C	0.29	0.60	0.01	0.08	0.01	0.18	0.21	0.11
rs7897156	C>T	O	0.33	0.28	0.03	0.03	0.01	0.07	0.02	0.02
rs4713599	A>C	O	0.10	0.63	0.79	0.26	0.63	0.79	0.42	0.92
rs2295778	C>G	C	0.29	0.59	0.22	0.90	0.38	0.22	0.42	0.59
rs7631002	T>C	O	0.11	0.53	0.91	0.27	0.70	0.83	0.18	0.47
rs1053639	A>T	O	0.17	0.34	0.31	0.56	0.28	0.35	0.52	0.31
rs3806408	C>A	C	0.30	0.15	0.46	0.05	0.14	0.73	0.13	0.32
rs3767199	G>T	O	0.09	0.73	0.19	0.24	0.13	0.92	0.33	0.94
rs10859966	C>T	O	0.42	0.31	0.75	0.68	0.98	0.47	0.84	0.72
rs1351383	A>C	C	0.39	0.27	0.33	0.15	0.87	0.43	0.16	0.19
rs3986354	C>T	O	0.46	0.16	0.68	0.34	0.77	0.12	0.66	0.47
rs2958153	G>A	O	0.29	0.77	0.86	0.87	0.94	0.98	0.88	0.96
rs479572	C>G	O	0.09	0.73	0.24	0.99	0.24	0.32	0.99	0.29
rs2284190	T>C	C	0.18	0.49	0.86	0.99	0.56	0.53	0.30	0.88
rs1064585	T>G	O	0.16	0.38	0.86	0.66	0.76	0.93	0.29	0.61
rs2819360	C>T	O	0.44	0.66	0.89	0.46	0.61	0.58	0.44	0.42
rs3755140	C>T	O	0.20	0.41	0.16	0.38	0.24	0.13	0.42	0.11
rs6671141	T>G	O	0.36	0.37	0.66	0.96	0.73	0.72	0.68	0.97
rs4324103	G>A	C	0.43	0.16	0.95	0.68	0.79	0.23	0.20	0.13
rs3812445	A>G	C	0.10	0.06	0.98	0.59	0.87	0.14	0.71	0.18
rs11823704	C>A	O	0.14	0.06	0.42	0.53	0.38	0.15	0.96	0.26
rs2070626	T>C	O	0.47	0.91	0.37	0.54	0.36	0.14	0.67	0.24
rs7942159	A>G	C	0.35	0.90	0.15	0.23	0.10	0.25	0.66	0.28
rs5750508	C>T	C	0.44	0.46	0.37	0.26	0.94	0.19	0.94	0.38
rs13624	T>C	O	0.14	0.41	0.09	0.99	0.36	0.58	0.28	0.94
rs3821321	G>A	O	0.42	0.41	0.64	0.90	0.69	0.21	0.34	0.16
rs4645287	A>C	O	0.57	0.40	0.27	0.81	0.64	0.70	0.97	0.81
rs1461957	G>T	O	0.17	0.35	0.83	0.82	0.80	0.99	0.64	0.88
rs3829051	T>C	O	0.31	0.34	0.39	0.24	0.94	0.20	0.12	0.88
rs734380	T>G	O	0.40	0.10	0.81	0.72	0.98	0.87	0.50	0.84
rs2282694	G>A	O	0.34	0.06	0.53	0.29	0.34	0.82	0.55	0.66
rs2748240	G>A	O	0.42	0.06	0.32	0.32	0.92	0.33	0.55	0.75
rs613924	T>C	C	0.42	0.97	0.15	0.99	0.33	0.50	0.79	0.76
rs2908543	T>A	O	0.33	0.97	0.05	0.41	0.06	0.40	0.34	0.28
rs2288834	G>A	O	0.40	0.56	0.55	0.34	0.35	0.76	0.99	0.84
rs3760109	G>T	O	0.20	0.48	0.34	0.38	0.63	0.27	0.55	0.51
rs11102516	G>A	C	0.42	0.38	0.83	0.30	0.64	0.82	0.41	0.54
rs7314442	G>A	C	0.22	0.38	0.36	0.86	0.41	0.99	0.41	0.78
rs9700928	C>A	O	0.43	0.27	0.73	0.52	0.57	0.66	0.76	0.65
rs3827723	A>G	O	0.36	0.23	0.43	0.41	0.88	0.29	0.33	0.82
rs2857681	C>G	C	0.33	0.11	0.64	0.17	0.38	0.46	0.61	0.68

rs2913861	T>C	C	0.20	0.07	0.47	0.43	0.38	0.92	0.43	0.68
rs2297480	G>T	O	0.22	0.06	0.62	0.87	0.75	0.81	0.69	0.98
rs3738037	C>A	O	0.08	0.06	0.89	0.74	0.99	0.91	0.83	0.87
rs12434539	A>G	O	0.34	0.05	0.66	0.63	0.58	0.58	0.57	0.50
rs10877028	G>T	C	0.18	0.97	0.13	0.33	0.30	0.17	0.44	0.32
rs866484	C>G	O	0.28	0.81	0.25	0.44	0.23	0.35	0.43	0.29
rs2913856	A>G	O	0.43	0.47	0.35	0.91	0.60	0.99	0.73	0.85
rs7071351	C>A	O	0.47	0.45	0.45	0.58	0.42	0.99	0.31	0.54
rs7648309	G>A	O	0.37	0.37	0.76	0.83	0.90	0.27	0.50	0.26
rs284573	C>G	O	0.14	0.28	0.82	0.90	0.88	0.41	0.47	0.62
rs7089806	C>G	O	0.15	0.17	0.81	0.75	0.76	0.59	0.88	0.71
rs2072236	C>T	O	0.12	0.10	0.23		0.23	0.64		0.64
rs7948073	T>G	O	0.24	0.08	0.15	0.42	0.16	0.59	0.25	0.38
rs7926547	C>G	O	0.39	0.06	0.20	0.30	0.17	0.67	0.63	0.59
rs17629719	T>G	O	0.19	0.98	0.77	0.07	0.44	0.48	0.53	0.42
rs7117343	C>G	O	0.28	0.83	0.58	0.32	0.38	0.84	0.28	0.52
rs12452627	G>A	O	0.15	0.82	0.88	0.28	0.66	0.56	0.72	0.67
rs2712429	C>A	O	0.14	0.52	0.84	0.51	0.97	0.60	0.44	0.48
rs549794	A>G	O	0.32	0.19	0.59	0.33	0.40	0.17	1.00	0.31
rs2403279	C>A	O	0.36	0.17	0.18	0.83	0.27	0.21	0.70	0.27
rs28407517	C>T	O	0.34	0.16	0.92	0.62	0.76	0.25	0.95	0.40
rs1043968	C>G	C	0.18	0.12	0.35	0.46	0.69	0.88	0.84	0.96
rs10876254	A>G	O	0.49	0.07	0.88	0.22	0.41	0.73	0.90	0.90
rs7073334	C>T	O	0.16	0.07	0.79	0.81	0.76	0.61	0.80	0.76
rs158366	C>G	O	0.36	0.98	0.67	0.18	0.70	0.89	0.38	0.73
rs6502051	A>C	C	0.09	0.69	0.95	0.99	0.71	0.93	0.98	0.76
rs1122731	G>A	O	0.20	0.59	0.74	0.99	0.98	0.45	0.99	0.26
rs10149569	A>C	O	0.11	0.49	0.82	0.99	0.78	0.45	0.99	0.39
rs10840108	T>C	O	0.35	0.45	0.36	0.13	0.15	0.40	0.32	0.28
rs2272532	A>G	O	0.22	0.43	0.68	0.81	0.67	0.96	0.60	0.87
rs13167616	G>C	O	0.13	0.29	0.97	0.71	0.87	0.40	0.41	0.69
rs34291864	G>T	O	0.18	0.25	0.58	0.87	0.59	0.28	0.35	0.22
rs10781517	C>T	O	0.14	0.24	0.33	0.31	0.67	0.41	0.50	0.65
rs2240275	G>A	C	0.41	0.23	0.65	0.45	0.96	0.71	0.32	0.44
rs2227562	G>A	O	0.34	0.17	0.68	0.47	0.52	0.12	0.16	0.07
rs2071391	A>G	O	0.47	0.16	0.44	0.40	0.34	0.58	0.63	0.53
rs2247322	T>A	O	0.35	0.15	0.94	0.68	0.80	0.50	0.53	0.43
rs3750997	A>C	O	0.47	0.14	0.70	0.94	0.77	0.31	0.17	0.14
rs12609068	A>T	C	0.28	0.11	0.84	0.46	0.63	0.93	0.26	0.58
rs580808	C>T	O	0.17	0.08	0.18	0.98	0.10	0.53	0.18	0.30
rs1976700	G>C	O	0.45	0.07	0.11	0.68	0.43	0.07	0.44	0.11
rs3809112	C>T	C	0.30	0.06	0.13	0.72	0.22	0.30	0.33	0.23

rs839763	T>C	O	0.14	0.05	0.85	0.64	0.99	0.47	0.44	0.80
rs625750	C>T	O	0.37	0.84	0.39	0.88	0.58	0.16	0.50	0.51
rs3827550	C>G	C	0.33	0.69	0.79	0.86	0.78	0.94	0.34	0.63
rs2954657	C>T	O	0.28	0.67	0.27	0.69	0.29	0.41	0.32	0.81
rs3849682	G>A	O	0.25	0.53	0.99	0.44	0.77	0.40	0.34	0.29
rs4645943	C>T	O	0.28	0.51	0.53	0.38	0.38	0.75	0.88	0.75
rs929953	A>G	O	0.35	0.34	0.76	0.32	0.78	0.30	0.31	0.22
rs10747783	C>T	O	0.27	0.26	0.61	0.85	0.78	0.64	0.81	0.81
rs1889532	G>A	O	0.14	0.25	0.47	0.42	0.69	0.55	1.00	0.60
rs3757672	T>C	O	0.11	0.10	0.41	0.74	0.41	0.69	0.16	0.48
rs629426	A>G	O	0.20	0.07	0.31	0.96	0.42	0.97	0.44	0.77
rs4135159	A>C	O	0.42	0.06	0.12	0.27	0.10	0.78	0.28	0.44
rs7366009	A>G	O	0.21	0.05	0.33	0.60	0.34	0.64	0.49	0.51
rs1567583	G>T	C	0.08	0.93	0.13	0.99	0.57	0.44	0.99	0.95
rs31726	A>G	C	0.43	0.91	0.31	0.44	0.28	0.11	0.90	0.34
rs3741440	C>T	O	0.07	0.71	0.88	0.99	0.86	0.09	0.99	0.12
rs2070912	A>G	O	0.13	0.63	0.88	0.99	1.00	0.84	0.38	0.74
rs3803800	G>A	C	0.34	0.48	0.91	0.51	0.69	0.31	0.36	0.75
rs1723285	G>A	O	0.14	0.47	0.29	0.99	0.23	0.37	0.79	0.37
rs2291617	G>T	C	0.27	0.22	0.20	0.75	0.28	0.35	0.63	0.36
rs2735784	A>G	O	0.43	0.88	0.79	0.90	0.80	0.37	0.84	0.48
rs2273913	T>C	C	0.47	0.84	0.70	0.48	0.85	0.96	0.20	0.42
rs1002193	C>G	O	0.34	0.84	0.41	0.22	0.21	0.13	0.28	0.10
rs4148879	G>A	C	0.17	0.84	0.43	0.24	0.30	0.34	0.24	0.23
rs3813570	T>C	O	0.48	0.79	0.93	0.11	0.29	0.54	0.49	0.96
rs2295685	G>A	O	0.24	0.71	0.29	0.97	0.38	0.62	0.63	0.56
rs228272	T>C	C	0.41	0.64	0.67	0.98	0.76	0.58	0.63	0.53
rs1461496	G>A	O	0.45	0.53	0.69	0.12	0.26	0.96	0.13	0.37
rs2079786	T>G	O	0.14	0.49	0.88	0.99	0.89	0.74	0.98	0.46
rs2070876	T>C	C	0.48	0.48	0.51	0.52	0.97	0.31	0.67	0.38
rs4735054	G>T	O	0.42	0.44	0.37	0.81	0.48	0.27	0.45	0.25
rs10424568	G>A	O	0.28	0.27	0.17	0.73	0.37	0.21	0.60	0.24
rs2845597	G>A	O	0.30	0.24	0.61	0.32	0.95	0.14	0.09	0.07
rs3806515	C>T	O	0.13	0.08	0.31	0.41	0.25	0.25	0.95	0.34
rs13169435	C>T	C	0.15	0.78	0.47	0.88	0.54	0.29	0.62	0.28
rs3789039	T>C	O	0.26	0.71	0.47	0.39	0.88	0.13	0.26	0.55
rs3809173	G>T	O	0.30	0.69	0.24	0.18	0.13	0.45	0.50	0.37
rs12901682	C>A	O	0.10	0.57	0.56	0.75	0.55	0.74	0.74	0.81
rs937215	T>C	O	0.19	0.87	0.47	0.58	0.42	0.74	0.75	0.70
rs2930975	C>G	O	0.19	0.79	0.27	0.60	0.26	0.08	0.70	0.10
rs17305311	T>C	O	0.38	0.58	0.67	0.54	1.00	0.93	0.66	0.88
rs2284992	G>T	C	0.32	0.46	0.48	0.72	0.49	0.31	0.36	0.24

rs17182246	G>C	O	0.35	0.42	0.35	0.35	0.88	0.42	0.09	0.75
rs7935835	G>A	C	0.23	0.31	0.07	0.25	0.21	0.08	0.58	0.18
rs3820312	A>C	O	0.37	0.06	0.54	0.56	0.90	0.75	0.93	0.86
rs8003631	T>C	C	0.38	0.86	0.62	0.85	0.79	0.24	0.30	0.71
rs3810288	C>G	C	0.30	0.68	0.80	0.55	0.65	0.16	0.59	0.18
rs2424993	C>G	O	0.45	0.60	0.18	0.86	0.45	0.08	0.84	0.22
rs1127155	G>A	O	0.38	0.39	0.38	0.30	0.25	0.95	0.49	0.69
rs2286620	T>C	O	0.22	0.22	0.43	0.58	0.61	0.90	0.34	0.66
rs2966449	C>T	C	0.22	0.11	0.47	0.69	0.71	0.43	0.75	0.45
rs2012124	C>T	O	0.41	0.97	0.18	0.86	0.41	0.30	0.28	0.90
rs10902227	C>T	C	0.36	0.73	0.52	0.93	0.62	0.05	0.51	0.09
rs9427715	T>G	O	0.51	0.69	0.47	0.70	0.83	0.78	0.21	0.33
rs534812	A>G	O	0.36	0.55	0.17	0.62	0.21	0.09	0.88	0.20
rs1467110	G>C	O	0.42	0.30	0.18	0.12	0.07	0.90	0.52	0.66
rs2303040	T>C	O	0.43	0.81	0.68	0.36	0.43	0.26	0.48	0.24
rs800351	T>C	O	0.45	0.70	0.71	0.89	0.88	0.92	0.61	0.72
rs7132224	A>G	O	0.52	0.42	0.37	0.37	0.27	0.83	0.14	0.29
rs3740883	C>T	O	0.17	0.10	0.93	0.21	0.62	0.56	0.33	0.40
rs9981301	G>C	C	0.50	0.94	0.64	0.71	0.94	0.56	0.59	0.99
rs12485058	A>G	O	0.24	0.19	0.56	0.93	0.64	0.91	0.53	0.91

Abbreviations: MAF, minor allele frequency; HWE-*P*, *P* for Hardy-Weinberg equilibrium test.

[a] O: SNP located on the gene, C: SNP closest to the gene.

[b] *P*-values were calculated using multivariate Cox proportional hazard models, adjusted for age, sex, smoking status, pathologic stage, and adjuvant therapy.

Supplementary Table S2. Overall survival and disease-free survival in the validation study.

	Overall survival					Disease free survival				
	Discovery cohort		Validation cohort			Discovery cohort		Validation cohort		
Polymorphism	HR(95% CI)[a]	*P*[a]	HR(95% CI)[a]	*P*[a]	P_H[b]	HR(95% CI)[a]	*P*[a]	HR(95% CI)[a]	*P*[a]	P_H[b]
rs17583										
Dominant	0.36(0.20-0.67)	0.001	0.46(0.23-0.90)	0.02	0.60	0.70(0.43-1.13)	0.15	0.62(0.40-0.96)	0.03	0.71
Recessive	1.02(0.42-2.47)	0.96	0.45(0.11-1.89)	0.27	0.34	0.62(0.28-1.38)	0.24	0.28(0.10-0.76)	0.01	0.22
Codominant	0.53(0.32-0.89)	0.02	0.52(0.30-0.92)	0.02	0.96	0.72(0.50-1.05)	0.09	0.60(0.43-0.85)	0.004	0.49
rs4751162										
Dominant	1.43(0.80-2.58)	0.23	1.04(0.51-2.12)	0.91	0.50	1.62(1.02-2.56)	0.04	1.50(0.97-2.31)	0.07	0.81
Recessive	1.86(0.24-14.65)	0.56	0.41(0.06-3.03)	0.38	0.30	0.90(0.12-6.75)	0.92	2.69(1.26-5.74)	0.01	0.32
Codominant	1.41(0.83-2.40)	0.21	0.92(0.52-1.62)	0.78	0.28	1.50(0.98-2.22)	0.06	1.52(1.09-2.14)	0.01	0.96
rs1529959										
Dominant	0.57(0.32-1.02)	0.06	1.59(0.71-3.58)	0.26	0.04	0.59(0.36-0.96)	0.03	0.92(0.58-1.46)	0.73	0.19
Recessive	1.13(0.61-2.09)	0.71	0.85(0.37-1.94)	0.69	0.59	1.14(0.69-1.87)	0.61	0.93(0.55-1.57)	0.78	0.58
Codominant	0.82(0.55-1.23)	0.34	1.12(0.71-1.78)	0.63	0.32	0.85(0.61-1.19)	0.33	0.94(0.70-1.27)	0.70	0.66
rs2601006										
Dominant	0.91(0.53-1.58)	0.75	0.62(0.30-1.25)	0.18	0.40	0.90(0.58-1.40)	0.65	0.87(0.56-1.33)	0.51	0.91
Recessive	0.37(0.11-1.21)	0.10	2.06(0.48-8.90)	0.33	0.07	0.34(0.14-0.86)	0.02	2.01(0.73-5.57)	0.18	0.01
Codominant	0.81(0.54-1.22)	0.30	0.74(0.40-1.40)	0.36	0.82	0.79(0.58-1.09)	0.15	0.96(0.65-1.41)	0.83	0.45
rs757228										
Dominant	0.62(0.35-1.11)	0.11	1.42(0.69-2.90)	0.34	0.08	0.61(0.38-0.96)	0.03	1.39(0.88-2.20)	0.16	0.01
Recessive	0.97(0.38-2.45)	0.94	1.47(0.64-3.36)	0.37	0.51	0.85(0.40-1.78)	0.67	1.65(0.94-2.90)	0.08	0.16
Codominant	0.74(0.46-1.19)	0.21	1.32(0.82-2.12)	0.25	0.09	0.71(0.49-1.03)	0.07	1.36(0.99-1.87)	0.06	0.01
rs11188272										
Dominant	0.72(0.42-1.23)	0.23	0.78(0.39-1.55)	0.48	0.86	0.92(0.60-1.43)	0.71	0.65(0.42-1.02)	0.06	0.28
Recessive	2.18(1.10-4.33)	0.03	0.95(0.33-2.71)	0.92	0.19	2.41(1.40-4.16)	0.00	1.45(0.81-2.62)	0.21	0.22
Codominant	1.05(0.70-1.57)	0.83	0.86(0.51-1.45)	0.57	0.55	1.24(0.90-1.71)	0.19	0.88(0.63-1.24)	0.47	0.15
rs4669										
Dominant	0.55(0.32-0.93)	0.03	1.01(0.49-2.07)	0.98	0.18	0.67(0.44-1.04)	0.07	0.99(0.63-1.55)	0.96	0.22
Recessive	1.15(0.58-2.25)	0.69	1.51(0.73-3.13)	0.27	0.59	1.26(0.74-2.12)	0.39	1.30(0.80-2.14)	0.29	0.93
Codominant	0.77(0.53-1.13)	0.19	1.17(0.74-1.87)	0.50	0.18	0.89(0.66-1.20)	0.45	1.09(0.81-1.47)	0.57	0.35
rs6049283										
Dominant	0.51(0.28-0.96)	0.04	0.86(0.41-1.83)	0.70	0.30	0.84(0.53-1.34)	0.46	1.08(0.68-1.70)	0.76	0.45
Recessive	2.02(0.70-5.86)	0.19	2.59(0.58-11.56)	0.21	0.79	1.96(0.87-4.42)	0.11	1.31(0.40-4.27)	0.65	0.58
Codominant	0.70(0.41-1.19)	0.19	1.01(0.53-1.94)	0.97	0.39	1.00(0.68-1.46)	0.99	1.09(0.73-1.63)	0.68	0.76
rs1205366										

Dominant	1.85(1.07-3.23)	0.03	0.60(0.28-1.29)	0.19	0.02	1.40(0.88-2.24)	0.16	0.88(0.56-1.39)	0.59	0.17
Recessive	1.12(0.26-4.88)	0.88	0(0-.)	0.99		1.78(0.63-5.04)	0.28	0.42(0.06-3.11)	0.40	0.21
Codominant	1.57(0.99-2.48)	0.05	0.57(0.29-1.14)	0.11	0.02	1.37(0.93-2.03)	0.11	0.86(0.57-1.30)	0.46	0.11
rs17528989										
Dominant	1.04(0.58-1.89)	0.89	0.53(0.23-1.22)	0.13	0.20	1.20(0.74-1.93)	0.47	0.80(0.49-1.30)	0.37	0.24
Recessive	2.19(0.67-7.21)	0.20	0.72(0.10-5.40)	0.75	0.35	3.61(1.43-9.13)	0.01	0.47(0.11-1.93)	0.29	0.02
Codominant	1.14(0.70-1.88)	0.60	0.60(0.29-1.24)	0.17	0.15	1.35(0.90-2.02)	0.15	0.79(0.52-1.19)	0.26	0.07
rs2425047										
Dominant	0.92(0.51-1.64)	0.78	0.67(0.31-1.44)	0.30	0.52	0.96(0.61-1.52)	0.87	1.08(0.67-1.74)	0.76	0.73
Recessive	3.28(1.25-8.62)	0.02	0(0-.)	0.99		1.95(0.77-4.94)	0.16	1.25(0.31-5.15)	0.75	0.61
Codominant	1.15(0.71-1.86)	0.56	0.65(0.31-1.37)	0.26	0.21	1.07(0.72-1.57)	0.75	1.08(0.71-1.66)	0.71	0.97
rs1136267										
Dominant	1.64(0.97-2.78)	0.07	1.03(0.54-1.98)	0.92	0.28	1.66(1.08-2.56)	0.02	1.01(0.67-1.53)	0.96	0.10
Recessive	1.00(0.22-4.53)	1.00	0.88(0.12-6.47)	0.90	0.92	1.00(0.24-4.19)	1.00	0.23(0.03-1.66)	0.15	0.24
Codominant	1.43(0.92-2.22)	0.11	1.01(0.57-1.81)	0.96	0.35	1.48(1.02-2.15)	0.04	0.91(0.63-1.30)	0.60	0.06
rs1803956										
Dominant	1.15(0.66-2.02)	0.62	1.04(0.52-2.08)	0.91	0.83	1.11(0.71-1.75)	0.64	0.91(0.59-1.40)	0.65	0.53
Recessive	2.19(1.16-4.13)	0.02	1.20(0.54-2.67)	0.65	0.25	1.78(1.04-3.04)	0.03	1.46(0.89-2.42)	0.14	0.60
Codominant	1.36(0.93-2.00)	0.11	1.08(0.68-1.71)	0.74	0.45	1.25(0.92-1.70)	0.16	1.08(0.80-1.46)	0.61	0.51
rs34087264										
Dominant	1.24(0.70-2.19)	0.47	1.07(0.53-2.18)	0.85	0.75	1.02(0.66-1.59)	0.93	1.04(0.66-1.64)	0.88	0.95
Recessive	0.51(0.22-1.16)	0.11	1.12(0.51-2.48)	0.78	0.18	0.43(0.21-0.88)	0.02	0.97(0.58-1.63)	0.90	0.07
Codominant	0.93(0.64-1.34)	0.69	1.07(0.67-1.70)	0.78	0.64	0.83(0.62-1.12)	0.22	1.01(0.75-1.35)	0.98	0.36
rs3896021										
Dominant	0.60(0.32-1.11)	0.10	1.28(0.55-2.98)	0.57	0.16	0.50(0.30-0.81)	0.01	0.82(0.51-1.31)	0.40	0.15
Recessive	0.96(0.53-1.75)	0.89	0.89(0.36-2.18)	0.79	0.89	0.70(0.42-1.17)	0.17	0.76(0.41-1.39)	0.37	0.84
Codominant	0.82(0.57-1.2)	0.30	1.06(0.63-1.79)	0.83	0.44	0.67(0.49-0.92)	0.01	0.83(0.60-1.16)	0.28	0.36
rs13817										
Dominant	1.80(1.00-3.23)	0.05	1.16(0.59-2.27)	0.67	0.33	1.03(0.66-1.62)	0.89	1.24(0.80-1.92)	0.33	0.56
Recessive	1.94(0.86-4.40)	0.11	0.34(0.08-1.42)	0.14	0.04	1.16(0.58-2.34)	0.68	1.13(0.59-2.15)	0.71	0.96
Codominant	1.63(1.07-2.48)	0.02	0.89(0.55-1.45)	0.63	0.07	1.05(0.75-1.48)	0.77	1.16(0.85-1.59)	0.36	0.68
rs2234216										
Dominant	1.03(0.60-1.78)	0.92	0.71(0.36-1.40)	0.33	0.40	0.87(0.57-1.34)	0.53	0.86(0.56-1.31)	0.48	0.97
Recessive	2.84(1.45-5.60)	0.00	1.52(0.62-3.73)	0.36	0.28	1.44(0.79-2.61)	0.23	1.05(0.54-2.04)	0.89	0.49
Codominant	1.37(0.91-2.07)	0.13	0.93(0.56-1.54)	0.77	0.24	1.02(0.74-1.41)	0.90	0.93(0.67-1.28)	0.65	0.69
rs6547618										
Dominant	0.74(0.41-1.34)	0.33	1.35(0.69-2.62)	0.38	0.19	1.00(0.63-1.59)	0.99	1.20(0.78-1.86)	0.41	0.58
Recessive	0.28(0.09-0.91)	0.03	0.92(0.27-3.17)	0.89	0.17	0.59(0.29-1.20)	0.15	0.75(0.34-1.65)	0.47	0.66
Codominant	0.66(0.43-1.02)	0.06	1.18(0.71-1.96)	0.52	0.09	0.88(0.64-1.21)	0.43	1.05(0.76-1.45)	0.77	0.45

rs6601178										
Dominant	2.33(1.22-4.43)	0.01	0.64(0.32-1.27)	0.20	0.01	1.85(1.12-3.05)	0.02	0.92(0.59-1.46)	0.73	0.04
Recessive	1.80(1.00-3.27)	0.05	1.24(0.54-2.83)	0.62	0.47	1.35(0.81-2.23)	0.25	1.51(0.90-2.55)	0.12	0.76
Codominant	1.66(1.16-2.38)	0.01	0.86(0.51-1.43)	0.55	0.04	1.40(1.04-1.87)	0.03	1.11(0.80-1.53)	0.54	0.29
rs2270625										
Dominant	2.08(1.2-3.58)	0.01	1.02(0.52-1.97)	0.96	0.10	1.26(0.81-1.95)	0.31	0.80(0.52-1.23)	0.31	0.15
Recessive	1.78(0.74-4.30)	0.20	1.79(0.54-5.97)	0.34	0.99	1.11(0.52-2.34)	0.79	0.60(0.19-1.91)	0.39	0.38
Codominant	1.67(1.13-2.47)	0.01	1.11(0.65-1.91)	0.70	0.23	1.16(0.84-1.60)	0.37	0.80(0.55-1.16)	0.24	0.14
rs17122154										
Dominant	1.26(0.68-2.36)	0.47	1.25(0.60-2.62)	0.55	0.99	1.76(1.04-3.00)	0.04	1.12(0.71-1.77)	0.63	0.21
Recessive	0.83(0.42-1.64)	0.59	2.44(1.21-4.92)	0.01	0.03	1.23(0.74-2.03)	0.43	1.05(0.61-1.82)	0.86	0.68
Codominant	1.03(0.71-1.50)	0.88	1.55(0.96-2.48)	0.07	0.18	1.31(0.97-1.77)	0.07	1.07(0.79-1.44)	0.67	0.35
rs2548551										
Dominant	1.46(0.79-2.70)	0.22	0.75(0.32-1.77)	0.51	0.22	1.12(0.68-1.84)	0.67	0.79(0.45-1.39)	0.42	0.36
Recessive	2.11(0.59-7.55)	0.25	0(0-.)	0.99		8.04(2.60-24.88)	0.00	2.01(0.27-15.05)	0.50	0.24
Codominant	1.43(0.87-2.34)	0.16	0.75(0.32-1.75)	0.50	0.20	1.32(0.85-2.07)	0.22	0.84(0.49-1.43)	0.52	0.20
rs10139029										
Dominant	1.92(1.05-3.49)	0.03	1.05(0.52-2.10)	0.90	0.20	1.08(0.65-1.77)	0.78	1.05(0.67-1.64)	0.84	0.93
Recessive	1.46(0.45-4.80)	0.53	1.30(0.17-9.92)	0.80	0.92	1.11(0.45-2.78)	0.82	1.13(0.35-3.68)	0.84	0.98
Codominant	1.58(1.00-2.50)	0.05	1.06(0.57-1.99)	0.86	0.32	1.06(0.73-1.56)	0.75	1.05(0.71-1.55)	0.81	0.97
rs665306										
Dominant	1.71(0.95-3.09)	0.08	1.23(0.63-2.40)	0.54	0.47	0.92(0.58-1.45)	0.71	1.26(0.82-1.94)	0.29	0.33
Recessive	2.08(0.91-4.76)	0.08	0.36(0.09-1.51)	0.16	0.04	1.07(0.53-2.17)	0.84	1.11(0.59-2.12)	0.74	0.94
Codominant	1.62(1.05-2.49)	0.03	0.94(0.58-1.51)	0.79	0.10	0.97(0.69-1.36)	0.85	1.16(0.85-1.59)	0.34	0.45
rs1010222										
Dominant	1.37(0.72-2.6)	0.34	0.87(0.42-1.81)	0.72	0.36	1.06(0.62-1.79)	0.84	1.14(0.72-1.81)	0.57	0.84
Recessive	3.28(0.76-14.16)	0.11	0(0-.)	0.99		4.55(1.35-15.32)	0.01	1.45(0.58-3.64)	0.43	0.14
Codominant	1.45(0.83-2.53)	0.20	0.74(0.40-1.37)	0.34	0.11	1.21(0.75-1.95)	0.44	1.15(0.80-1.67)	0.45	0.87
rs2271446										
Dominant	1.79(1.02-3.14)	0.04	1.13(0.58-2.19)	0.72	0.30	1.52(0.97-2.40)	0.07	1.11(0.72-1.71)	0.63	0.33
Recessive	1.62(0.70-3.72)	0.26	0.55(0.13-2.28)	0.41	0.20	1.30(0.61-2.76)	0.50	0.77(0.36-1.68)	0.51	0.34
Codominant	1.48(1.01-2.17)	0.05	0.97(0.59-1.61)	0.91	0.19	1.32(0.95-1.84)	0.10	1.01(0.74-1.39)	0.95	0.25
rs3825625										
Dominant	1.90(1.05-3.44)	0.03	1.53(0.74-3.13)	0.25	0.65	1.49(0.92-2.43)	0.11	1.02(0.62-1.66)	0.95	0.28
Recessive	1.66(0.22-12.71)	0.63	0(0-.)	0.99		1.62(0.38-6.95)	0.51	1.45(0.19-11.05)	0.72	0.93
Codominant	1.75(1.04-2.97)	0.04	1.45(0.72-2.9)	0.30	0.67	1.44(0.93-2.22)	0.10	1.03(0.65-1.65)	0.90	0.30
rs7513326										
Dominant	1.64(0.88-3.05)	0.12	0.87(0.44-1.74)	0.69	0.18	1.16(0.72-1.87)	0.55	1.12(0.70-1.81)	0.63	0.92
Recessive	2.37(1.24-4.56)	0.01	0.75(0.26-2.13)	0.59	0.07	1.74(1.02-2.96)	0.04	1.62(0.94-2.79)	0.08	0.85

Codominant	1.66(1.11-2.47)	0.01	0.86(0.51-1.44)	0.56	0.05	1.27(0.93-1.75)	0.14	1.25(0.89-1.75)	0.20	0.95
rs772254										
Dominant	2.03(1.12-3.68)	0.02	0.75(0.36-1.55)	0.43	0.04	1.51(0.94-2.42)	0.09	0.68(0.43-1.09)	0.11	0.02
Recessive	1.59(0.55-4.62)	0.39	0(0-.)	0.99		1.60(0.67-3.79)	0.29	1.35(0.42-4.40)	0.62	0.82
Codominant	1.61(1.05-2.48)	0.03	0.71(0.36-1.39)	0.31	0.04	1.38(0.97-1.98)	0.08	0.76(0.50-1.15)	0.19	0.03
rs11668444										
Dominant	1.74(0.93-3.25)	0.09	1.06(0.53-2.13)	0.87	0.30	1.25(0.76-2.04)	0.38	0.63(0.38-1.03)	0.06	0.05
Recessive	3.46(1.21-9.94)	0.02	2.13(0.26-17.43)	0.48	0.69	1.75(0.62-4.88)	0.29	3.42(1.14-10.26)	0.03	0.38
Codominant	1.74(1.08-2.82)	0.02	1.11(0.59-2.11)	0.75	0.27	1.26(0.84-1.88)	0.27	0.76(0.49-1.19)	0.24	0.10
rs1279738										
Dominant	3.40(1.63-7.09)	0.00	1.01(0.51-2.01)	0.97	0.02	1.92(1.12-3.30)	0.02	0.79(0.51-1.21)	0.28	0.01
Recessive	2.48(1.34-4.60)	0.00	1.83(0.82-4.08)	0.14	0.56	1.74(1.02-2.97)	0.04	1.48(0.85-2.58)	0.16	0.68
Codominant	2.09(1.42-3.08)	0.00	1.22(0.75-1.98)	0.43	0.09	1.56(1.13-2.15)	0.01	0.99(0.72-1.37)	0.95	0.05
rs2075588										
Dominant	1.63(0.93-2.85)	0.09	0.62(0.32-1.18)	0.14	0.03	1.26(0.81-1.95)	0.31	0.67(0.44-1.03)	0.07	0.04
Recessive	1.69(0.66-4.33)	0.27	0.29(0.04-2.12)	0.22	0.12	2.42(1.07-5.47)	0.03	0.51(0.21-1.28)	0.15	0.01
Codominant	1.50(0.98-2.30)	0.06	0.61(0.35-1.07)	0.08	0.01	1.36(0.94-1.97)	0.11	0.69(0.49-0.98)	0.04	0.01
rs3806192										
Dominant	1.24(0.70-2.21)	0.46	0.99(0.50-1.95)	0.97	0.62	1.81(1.13-2.9)	0.01	0.76(0.48-1.19)	0.23	0.01
Recessive	0(0-.)	0.99	1.90(0.40-8.97)	0.42		0(0-.)	0.98	1.10(0.33-3.71)	0.88	
Codominant	1.17(0.68-2.03)	0.57	1.07(0.59-1.95)	0.82	0.83	1.59(1.03-2.44)	0.03	0.81(0.54-1.22)	0.31	0.03
rs843346										
Dominant	0.98(0.55-1.75)	0.94	1.11(0.57-2.15)	0.76	0.78	0.90(0.57-1.42)	0.65	0.99(0.64-1.52)	0.95	0.77
Recessive	0.08(0.01-0.58)	0.01	0.68(0.23-2.01)	0.49	0.06	0.32(0.13-0.79)	0.01	0.92(0.46-1.85)	0.82	0.07
Codominant	0.70(0.46-1.06)	0.09	0.97(0.61-1.56)	0.90	0.31	0.77(0.55-1.06)	0.11	0.98(0.71-1.35)	0.88	0.30
rs1242095										
Dominant	1.19(0.67-2.12)	0.55	0.95(0.48-1.86)	0.87	0.62	1.53(0.96-2.44)	0.07	0.89(0.58-1.36)	0.59	0.09
Recessive	2.69(1.13-6.38)	0.03	0.92(0.40-2.10)	0.84	0.08	2.19(1.18-4.07)	0.01	0.79(0.46-1.39)	0.42	0.02
Codominant	1.40(0.89-2.21)	0.15	0.95(0.61-1.49)	0.83	0.24	1.53(1.09-2.13)	0.01	0.89(0.67-1.19)	0.42	0.02
rs2292807										
Dominant	1.13(0.60-2.14)	0.70	1.00(0.51-1.94)	0.99	0.79	1.29(0.79-2.12)	0.31	0.57(0.37-0.88)	0.01	0.02
Recessive	0.43(0.20-0.92)	0.03	0.86(0.37-1.96)	0.71	0.23	0.66(0.37-1.18)	0.16	0.70(0.40-1.23)	0.22	0.89
Codominant	0.82(0.56-1.19)	0.29	0.96(0.62-1.47)	0.83	0.59	0.98(0.73-1.31)	0.88	0.70(0.52-0.94)	0.02	0.11
rs6679										
Dominant	1.29(0.71-2.36)	0.40	2.15(0.93-4.96)	0.07	0.33	1.66(1.01-2.73)	0.04	1.11(0.70-1.76)	0.66	0.24
Recessive	0.83(0.38-1.80)	0.64	0.54(0.23-1.30)	0.17	0.47	1.31(0.72-2.38)	0.38	0.83(0.50-1.37)	0.47	0.25
Codominant	1.07(0.72-1.57)	0.75	1.07(0.70-1.63)	0.77	1.00	1.35(0.98-1.85)	0.06	0.98(0.74-1.29)	0.88	0.13
rs11809443										
Dominant	0.82(0.46-1.46)	0.50	1.05(0.53-2.09)	0.89	0.59	0.96(0.61-1.52)	0.87	1.05(0.67-1.64)	0.84	0.78

Recessive	0.23(0.05-1.00)	0.05	2.98(0.68-13.04)	0.15	0.02	0.50(0.15-1.66)	0.25	1.84(0.57-5.91)	0.31	0.13
Codominant	0.72(0.46-1.13)	0.15	1.18(0.64-2.18)	0.59	0.20	0.90(0.62-1.30)	0.56	1.10(0.73-1.65)	0.65	0.47
rs2271541										
Dominant	0.55(0.32-0.95)	0.03	0.96(0.48-1.91)	0.91	0.21	0.64(0.41-1.02)	0.06	0.70(0.45-1.08)	0.10	0.78
Recessive	1.77(0.88-3.54)	0.11	0.59(0.21-1.67)	0.32	0.09	1.50(0.85-2.66)	0.17	0.71(0.38-1.32)	0.28	0.08
Codominant	0.87(0.57-1.32)	0.51	0.86(0.53-1.39)	0.53	0.97	0.91(0.64-1.28)	0.57	0.76(0.55-1.04)	0.08	0.45
rs3813521										
Dominant	0.47(0.27-0.84)	0.01	0.61(0.31-1.19)	0.15	0.56	0.74(0.47-1.15)	0.18	1.06(0.70-1.62)	0.77	0.25
Recessive	0.27(0.06-1.17)	0.08	0.26(0.04-1.90)	0.18	0.98	0.55(0.22-1.40)	0.21	1.14(0.57-2.31)	0.71	0.22
Codominant	0.51(0.32-0.83)	0.01	0.61(0.34-1.07)	0.08	0.64	0.75(0.52-1.07)	0.11	1.06(0.78-1.46)	0.70	0.16
rs7897156										
Dominant	1.97(1.08-3.57)	0.03	1.02(0.52-1.99)	0.96	0.15	1.54(0.97-2.45)	0.07	0.93(0.60-1.44)	0.73	0.12
Recessive	2.37(1.11-5.07)	0.03	1.28(0.39-4.22)	0.69	0.39	2.16(1.12-4.14)	0.02	1.56(0.71-3.45)	0.27	0.53
Codominant	1.76(1.17-2.64)	0.01	1.06(0.62-1.79)	0.83	0.13	1.51(1.08-2.12)	0.02	1.03(0.72-1.47)	0.89	0.13

[a] Hazard ratios (HRs), 95% confidence intervals (CIs) and corresponding *P*-values were calculated using multivariate Cox proportional hazard models, adjusted for age, gender, smoking status, pathologic stage and adjuvant therapy.

[b] P_H, *P* for heterogeneity test.

Supplementary Table S3. Primers used in this study

Assay	Gene	Primers
Promoter assay	rs17583 forward	5'-CCGCTCGAGAACCCCTCCCTCTTGCAAGC -3'
	rs17583 reverse	5'-CCCAAGCTTGGTCATCCTTCCGCATCTCC -3'
	GLRX3 pro forward	5'-CCGCTCGAGGGACTGAATTGGAAGCACCC-3'
	GLRX3 pro reverse	5'-CATGCCATGGCGCCAGACAGAAGCAATCCA-3'
	rs4751162 forward	5'-CGGGATCCGTGCAAGTCTCCCCATTACC-3'
	rs4751162 reverse	5'-ACGCGTCGACAGACCCAGGCTCCCTAAAAC-3'
qRT-PCR	*CAPN1* forward	5'-ATTTCCAGCTGTGGCAATTT-3'
	CAPN1 reverse	5'-CTCCAGAACTCGTTGCCTTC-3'
	LINC00959 forward	5'-TGCTCCCATCCCTGCCATGT-3'
	LINC00959 reverse	5'-AAGACAGGAATCTCGGGTGGGC-3'
	GLRX3 forward	5'-AGATGAACGAAGTTATGGCAGAG-3'
	GLRX3 reverse	5'-ACAGAAAAGTGGGAACAGAGC-3'
	β-actin forward	5'-TTGTTACAGGAAGTCCCTTGCC-3'
	β-actin reverse	5'-ATGCTATCACCTCCCCTGTGT-3'
ChIP-qPCR	rs17583 forward	5'-GATTCTCCCTAGCACCCGCT-3'
	rs17583 reverse	5'-TGCCGGCATAGCCATTCTGG-3'
	rs4751162 forward	5'-TACTCGTCCCCCAACACCCA-3'
	rs4751162 reverse	5'-GGCCGAGACTCATTGCAGGA-3'
